# Urinary NGAL Measured after the First Year Post Kidney Transplantation Predicts Changes in Glomerular Filtration over One-Year Follow-Up

**DOI:** 10.3390/jcm10010043

**Published:** 2020-12-25

**Authors:** Małgorzata Kielar, Paulina Dumnicka, Agnieszka Gala-Błądzińska, Alina Będkowska-Prokop, Ewa Ignacak, Barbara Maziarz, Piotr Ceranowicz, Beata Kuśnierz-Cabala

**Affiliations:** 1 St. Louis Regional Children’s Hospital, Medical Diagnostic Laboratory with a Bacteriology Laboratory, Strzelecka 2 St., 31-503 Kraków, Poland; gkielar@tlen.pl; 2 Jagiellonian University Medical College, Faculty of Pharmacy, Department of Medical Diagnostics, 30-688 Kraków, Poland; paulina.dumnicka@uj.edu.pl; 3 Medical College of Rzeszów University, Institute of Medical Sciences, Kopisto 2A Avn., 35-310 Rzeszów, Poland; agala.edu@gmail.com; 4 Jagiellonian University Medical College, Faculty of Medicine, Department of Nephrology, Jakubowskiego 2 St., 30-688 Kraków, Poland; alina.betkowska-prokop@uj.edu.pl (A.B.-P.); ewa.igncak@uj.edu.pl (E.I.); 5 Jagiellonian University Medical College, Faculty of Medicine, Department of Diagnostics, Kopernika 15A St., 31-501 Kraków, Poland; mbmaziar@cyf-kr.edu.pl; 6 Jagiellonian University Medical College, Faculty of Medicine, Department of Physiology, Grzegórzecka 16 St., 31-531 Kraków, Poland

**Keywords:** kidney allograft, neutrophil gelatinase-associated lipocalin, matrix metalloproteinase 9-neutrophil gelatinase-associated lipocalin complex, glomerular filtration rate, urinary tract infections

## Abstract

Currently, serum creatinine and estimated glomerular filtration rate (eGFR) together with albuminuria or proteinuria are laboratory markers used in long-term monitoring of kidney transplant recipients. There is a need for more sensitive markers that could serve as early warning signs of graft dysfunction. Our aim was to assess the urinary concentrations of neutrophil gelatinase-associated lipocalin (NGAL) as a predictor of changes in kidney transplant function after the first year post-transplantation. We prospectively recruited 109 patients with functioning graft at least one year after the transplantation, with no acute conditions over the past three months, during their control visits in kidney transplant ambulatory. Urinary NGAL measured on recruitment was twice higher in patients with at least 10% decrease in eGFR over 1-year follow-up compared to those with stable or improving transplant function. Baseline NGAL significantly predicted the relative and absolute changes in eGFR and the mean eGFR during the follow-up independently of baseline eGFR and albuminuria. Moreover, baseline NGAL significantly predicted urinary tract infections during the follow-up, although the infections were not associated with decreasing eGFR. Additionally, we assessed urinary concentrations of matrix metalloproteinase 9—NGAL complex in a subgroup of 77 patients and found higher levels in patients who developed urinary tract infections during the follow-up but not in those with decreasing eGFR. High urinary NGAL in clinically stable kidney transplant recipients beyond the first year after transplantation may be interpreted as a warning and trigger the search for transient or chronic causes of graft dysfunction, or urinary tract infection.

## 1. Introduction

Renal transplantation is the best therapeutic option for patients with end-stage kidney disease. Both donor—and recipient-related factors are associated with kidney graft function, including metabolic and immunological factors. Acute or chronic rejection, recurrent or de novo nephropathies, side effects of immunosuppressive drugs, comorbidities (including infections), and age-related decline in renal function adversely affect long-term survival of both kidney graft and transplant recipient. According to 2009 Kidney Disease: Improving Global Outcomes (KDIGO) guidelines [1], the monitoring of kidney transplant recipients is based on physical examination, urine volume, the assessment of albuminuria or proteinuria, serum creatinine measurements, glomerular filtration rate (GFR) estimation based on serum creatinine, and the ultrasound imaging. Kidney transplant biopsy and histopathological examination allow precise diagnosis of graft injury. Although protocol-specified biopsies are performed in some centers [2], the biopsy procedure is invasive and is associated with possible adverse events. Therefore, most centers only use graft biopsy in the patients with worsening renal function i.e., suspected acute kidney rejection episodes or a chronic elevation in creatinine and/or the onset of persistent proteinuria (indication or “for cause” biopsies), as recommended by the KDIGO guidelines [1]. This allows the diagnosis of kidney graft injury. However, the indication for graft biopsy based on the clinical assessment, including serum creatinine, eGFR, and albuminuria (proteinuria) may in some cases be controversial.

The studies using protocol biopsies reveal the signs of chronic rejection in a significant proportion of cases without clinical signs of decreasing renal function [3,4]. Still, even subclinical chronic rejection leads to a decrease in graft survival over long-term time periods [4]. There is a need for novel markers that may be used in routine monitoring of patients and would provide earlier warning signal as compared to currently recommended markers used in clinical practice, i.e., serum creatinine, eGFR and albuminuria or proteinuria. Specifically, serum creatinine concentrations, and thus eGFR, may be influenced by extra-renal factors, leading to the intra-individual biological variability that has been estimated for about 5–8% [5,6,7,8].

In the last 15 years, neutrophil gelatinase-associated lipocalin (NGAL) became one of the most extensively studied marker of acute kidney injury (AKI) [9,10]. NGAL is a 25 kDa protein that has been originally isolated from the secondary granules of activated neutrophils [11]. It is also produced by hepatocytes, the cells of alimentary and respiratory tracts, the cells of immune system, and, notably, by the epithelial cells of distal renal tubule [12,13,14]. Both toxic and ischemic kidney injury leads to increased excretion of NGAL in urine which may be noted as soon as two hours from the initial insult [12,14,15]. Three molecular forms of NGAL have been found in urine, namely the NGAL monomer that has been associated with distal tubular cells injury, the NGAL homodimer that has been associated with the presence of neutrophils in urine and urinary tract infections, and the least studied matrix metalloproteinase 9 (MMP 9)-NGAL heterodimer [13,16]. NGAL has been assigned the protective role during acute tubular injury, increasing autophagy in the distal tubular cells, inhibiting apoptosis and inducing regeneration [17,18,19]. More recently, NGAL has been studied as a marker of chronic kidney disease (CKD), including diabetic kidney disease [20,21], and as a marker of cardiovascular risk [22,23]. In kidney transplantation, NGAL (measured early post-transplant) has been extensively studied as a predictor of delayed graft function [13,24,25,26]. Also, there are several reports on urinary NGAL as a predictor of acute kidney injury later after transplantation [27,28] and a graft loss after acute kidney injury [29]. However, the diagnostic value of NGAL in kidney transplant patients following the first year after transplantation, in relation to chronic processes leading to a gradual decrease in kidney allograft function, has not been extensively studied [30,31]. Moreover, clinical utility of the published findings is hindered by the use of diverse laboratory methods for urine NGAL measurements, which does not allow directly comparing the measured concentrations and the proposed cut-off values.

Our aim was to assess the concentrations of urinary NGAL with an automated laboratory method in kidney transplant recipients at least one year after transplantation and to study their association with changes in kidney function observed during one-year follow-up. Considering the intra-individual variability of serum creatinine and eGFR that is observed in clinical practice among chronic kidney disease patients, we compared the subgroups of patients with and without at least 10% decrease in eGFR over a 1-year follow-up. Additionally, we performed an exploratory analysis regarding the urinary concentrations of MMP 9-NGAL complex in a subgroup of studied patients.

## 2. Methods

### 2.1. Study Design

This was a prospective observational study. Patients were recruited in the ward dedicated to care of kidney transplant recipients, in the Chair and Department of Nephrology, University Hospital, Kraków, Poland between May and July 2019. The inclusion criteria were as follows: age of at least 18 years, at least one year from transplantation, functioning kidney transplant with eGFR at least 15 mL/min/1.73 m^2^, and no acute kidney injury fulfilling the definition of KDIGO 2011 [32] or any condition requiring hospital treatment during at least three months before the inclusion into the study. Patients with the signs or symptoms of infection, including urinary tract infection at baseline were excluded from the study.

On recruitment, patients signed an informed consent for the study. They underwent detailed history and physical examination. Urine and venous blood samples were collected for the laboratory tests in the morning of the day of the study visit. The baseline clinical data and the results of routine laboratory tests on enrollment were recorded. The data regarding transplantation procedure (date of transplantation, deceased or living donor, first or second transplant, induction therapy, cold and warm ischemia time, delayed graft function) and the primary cause of kidney disease were based on the available medical records. The data on pretransplant panel reactive antibodies (PRA) and donor/recipient human leukocyte antigens (HLA) mismatches were based on transplantation protocols available for the patients who had undergone transplantation procedure in University Hospital, Kraków, Poland. In September 2020, the follow-up data were collected based on medical records of the patients who remained in control in the kidney transplant recipients’ ambulatory, including the clinical course during the follow-up, the serum creatinine concentrations obtained during all control visits, and the results of laboratory tests performed at the last visit of a patient in the kidney transplant recipients’ ambulatory.

The study protocol was approved by the Jagiellonian University Bioethical Committee (approval no 1072.6120.46.2019 issued on 28 February 2019).

### 2.2. Laboratory Tests

The blood and urine samples for laboratory tests were collected in the morning hours after an overnight fast. Routine laboratory test included complete blood count performed in K_2_EDTA-anticoagulated samples, biochemical and immunochemical tests (serum creatinine, triglycerides, total cholesterol, uric acid, albumin, C-reactive protein, and glucose), and urinalysis. In addition, urine protein, albumin, NGAL and creatinine concentrations were assessed in the samples obtained at the initial study visit. Excess serum and urine samples were aliquoted and frozen in −80 °C within 2 h from samples’ collection. Within two months, the thawed samples were centrifuged and the supernatant was analyzed for NGAL and MMP 9-NGAL complex. Urine NGAL was assessed with chemiluminescence microparticle immunoassay (Architect urine NGAL) on Abbott Architect analyzer (Abbott Laboratories, Chicago, IL, USA). The limit of detection for the test was estimated for 0.7 ng/mL by the manufacturer; the intra- and inter-assay precision were ≤5.2% and ≤6.7%, respectively. The tests were performed in Diagnostic Department of Hospital University, Krakow, Poland, on the day of samples’ collection. MMP 9-NGAL complex was assessed in series in duplicate wells per sample using Quantikine ELISA Human MMP-9/NGAL Complex Immunoassay (R&D Systems, McKinley Place, MN, USA). The minimum detectable dose of human MMP 9-NGAL was 0.013 ng/mL. According to the manufacturer of the test, the mean urine concentrations in healthy volunteers was 0.44 ng/mL (ranged from non-detectable to 0.67 ng/mL). The measurements were performed in the Department of Diagnostics, Chair of Clinical Biochemistry, Jagiellonian University Medical College, Krakow, Poland.

### 2.3. Statistical Analysis

The number of patients and the percentage of the respective group were reported for categories. The contingency tables were analyzed with Pearson’s chi-squared test. The mean ± standard deviation (SD) or median with lower and upper quartile (Q1; Q3) were reported for quantitative variables with or without normal distribution, respectively. The variables’ distributions were assessed for normality with the Shapiro-Wilk’s test. The groups were compared using t-test or Mann-Whitney’s test, depending on the variables’ distributions. Time-related changes were assessed with the Wilcoxon’s matched pairs test as the appropriate variables’ distributions differed from normal. Simple correlations were analyzed with the Pearson’s correlation coefficient, calculated using log-transformed variables in case of right-skewed distribution. Simple correlations between urinary NGAL, NGAL/creatinine and the number of mismatched HLA were analyzed with the Spearman rank coefficient. Multiple linear regression was used to identify the independent predictors of eGFR changes and eGFR based on mean creatinine during the follow-up period. The regression models included the independent variables that were significantly associated with at least one dependent variable in simple analysis, and were additionally adjusted for time from kidney transplantation (because of the considerable diversity of the time from transplantation in the studied group). Sex-adjusted logistic regression was used to assess the studied laboratory markers as the predictors of urinary tract infections during follow-up. Receiver operating characteristics (ROC) curve analysis was used to assess and compare the diagnostic accuracy of urine NGAL and albumin concentrations (raw and corrected to urine creatinine) for the prediction of at least 10% decrease in eGFR values over the follow-up period. The cut-off values were selected using maximum Youden index. All tests were two-tailed; the results were considered significant at *p*-value < 0.05. Statistica 12 (StatSoft, Tulsa, OK, USA) and Statistica 13 software (Tibco Software Inc., Tulsa, OK, USA) were used for computation.

## 3. Results

### 3.1. Baseline Characteristics of Patients

We recruited 109 adult kidney transplant recipients (43 women and 66 men, aged between 19 and 78 years). The patients were recruited in a single center, the Chair and Department of Nephrology, University Hospital in Kraków, Poland, during the control ambulatory visits. The time from transplantation ranged from 1 year to 22 years, with a median of 7 years. According to 2012 KDIGO guidelines on chronic kidney disease [33], there were 5 (5%) patients in G1T, 29 (27%) in G2T, 22 (20%) in G3aT, 42 (39%) in G3bT, and 11 (10%) in G4T stage as based on baseline eGFR. Forty-nine (45%) patients had normal to mildly increased albuminuria (A1), 39 (36%) moderately increased albuminuria (A2) and 21 (19%) severely increased albuminuria (A3).

The follow-up data were collected during planned control visits over a period of up to 16 months from the enrollment. After a median 12.4-month observation (range 3.2 to 15.4 months; Q1; Q3: 11.2; 13.3 months), 10% or higher decrease in eGFR (i.e., final eGFR ≤90% of initial value) was noted in 30 patients (28%; Table 1). The baseline clinical characteristics of patients with ≥10% decrease in eGFR did not differ significantly from the rest of the group, except for higher maximum panel reactive antibodies’ percentage in patients with decreasing eGFR (Table 1).

Urinary albumin and albumin to creatinine ratio (uACR) were significantly higher in patients with ≥10% decrease in eGFR over a follow-up (Table 2). Also, these patients had lower initial eGFR values. Slightly but significantly lower serum albumin and blood hemoglobin concentrations were associated with ≥10% eGFR decrease. Both urinary NGAL concentration and NGAL to creatinine ratio were higher in patients with decreasing eGFR (Table 2).

### 3.2. Changes in eGFR over a Follow-Up Period

**Figure 1:**
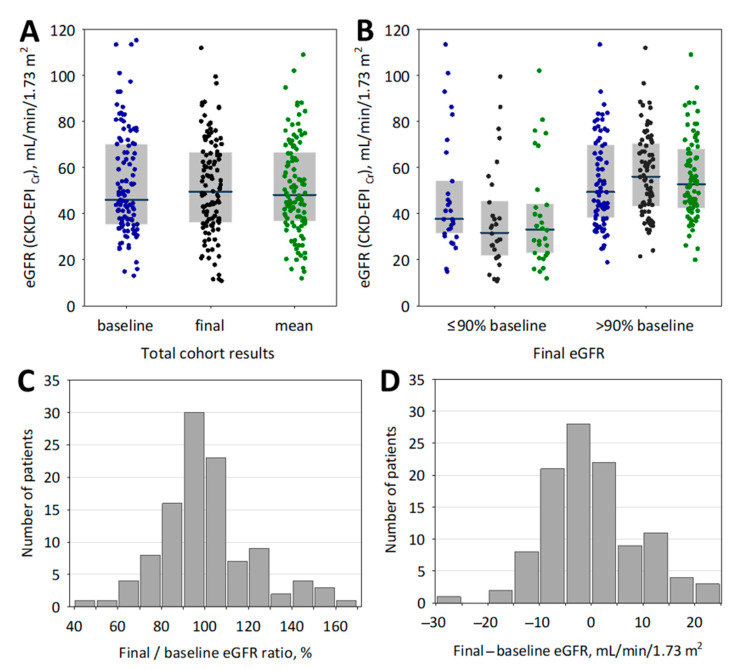
Baseline (blue points), final (black points) eGFR values and eGFR values based on mean creatinine during follow-up (“mean”; green points) in the whole studied cohort of kidney transplant recipients (**A**) and in the subgroups defined by final to baseline eGFR ratio below or above 90% of baseline value (**B**). Data on panels A and B are shown as median (central line), interquartile range (box) and raw data (points). The histograms showing percentage (**C**) and absolute (**D**) changes in eGFR values over the follow-up period in the whole studied group of kidney transplant recipients.

During the follow-up, we observed both increases and decreases in eGFR values (Figure 1). In the whole studied cohort, this resulted in no difference between initial and final eGFR values (*p* = 0.8; Figure 1A). In patients with final to baseline eGFR ratio ≤90%, the eGFR values decreased by a median of 19% (Q1; Q3: 16; 25%) or by 9 mL/min/1.73 m^2^ (Q1; Q3: 6; 14 mL/min/1.73 m^2^). On the contrary, we observed a significant increase in eGFR values in the subgroup with final eGFR >90% of baseline eGFR (*p* < 0.001; Figure 1B), resulting in a highly significant difference in final eGFR values between the studied subgroups: median final eGFR was 30 mL/min/1.73 m^2^ in patients with final eGFR ≤90% of the baseline values, and 56 mL/min/1.73 m^2^ in patients with final eGFR >90% baseline (*p* < 0.001).

Moreover, we analyzed eGFR values calculated based on the arithmetic mean of all serum creatinine measurements performed during the follow-up (excluding the initial eGFR value recorded on enrollment; Figure 1A,B). The median number of visits during the follow-up period was 4 (range 1–7; Q1; Q3: 3; 5) and did not differ significantly between patients with ≥10% decrease in eGFR and the rest of the group (*p* = 0.2). The median eGFR based on mean creatinine during the follow-up period was 48 (Q1; Q3: 37; 68) mL/min/1.73 m^2^ and was significantly lower among patients with ≥10% decrease in eGFR as compared to the rest of the group (median 33 and 53 mL/min/1.73 m^2^, respectively; *p* < 0.001; Figure 1B).

During the studied period, the attending physicians of kidney transplant ambulatory diagnosed the clinically relevant and sustained decrease in kidney transplant function in nine patients, i.e., 30% of the group with ≥10% decrease in eGFR. It was attributed to chronic rejection in two patients, recurrence of glomerulonephritis in one patient, urological complications with recurrent urinary tract infections in one patient, and due to unknown causes in the remaining five patients (two of them refused to undergo kidney transplant biopsy). Two of these patients progressed to stage G5 CKD and were referred to hemodialysis treatment. Further four patients were diagnosed with urinary tract infection, two with other infections, one with recurrent glomerulonephritis, two with cardiovascular complications, and one with adverse drug reaction that were recognized as resulting in a transient decrease of the transplant function. In the remaining 11 patients, there was no entry in their medical records about the (suspected) cause of eGFR decrease in medical records.

### 3.3. Associations between Urinary NGAL Concentrations and the Baseline Characteristics of Studied Patients

The baseline urinary concentrations of NGAL (log-transformed: R = −0.06; *p* = 0.5) and NGAL to creatinine ratios (log-transformed: R = −0.09; *p* = 0.4) did not correlate with the baseline eGFR values either in the total cohort and in the subgroups defined by final to baseline eGFR ratio below or above 90% of the baseline value. Urine NGAL concentration and NGAL to creatinine ratio were significantly correlated with urine albumin and uACR at baseline (R equaled from 0.36 to 0.48 for log-transformed variables; *p* ≤ 0.001). Urinary NGAL and NGAL/creatinine were not associated with age (*p* > 0.7), sex (*p* > 0.1), or time from transplantation (*p* > 0.5). Among the patients who had undergone transplantation procedure in our center, there was a marginally significant positive correlation between the urine NGAL concentration and the number of donor/recipient HLA mismatches (Spearman R = 0.31; *p* = 0.049). Other variables listed in Table 1 showed no associations with urinary NGAL and NGAL/creatinine.

### 3.4. Associations between Clinical and Laboratory Characteristics and the Changes in eGFR over a Follow-Up Period

Baseline eGFR values, urinary albumin, and albumin to creatinine ratios, urinary NGAL and NGAL to creatinine ratios (but neither MMP 9-NGAL complex concentrations nor MMP 9-NGAl/creatinine ratios), as well as serum uric acid and albumin concentrations were significantly correlated with either the percentage or the absolute changes in eGFR values during the observation (Table 3). Moreover, baseline eGFR, urinary albumin concentration, uACR, urinary NGAL concentration and NGAL/creatinine ratio as well as serum uric acid correlated significantly with eGFR values based on the mean of all serum creatinine results obtained during the follow-up period (Table 3). No significant correlations were observed between the percentage or absolute changes in eGFR and the clinical characteristics, including age, time from transplantation, cold or warm ischemia time, blood pressure values, or daily diuresis (*p* > 0.1 in all cases). Neither the ratio of follow-up to baseline eGFR (R = 0.15; *p* = 0.1) nor the absolute difference between the follow-up and the baseline eGFR values (R = 0.14; *p* = 0.1) correlated with the length of observation. Also, neither the changes in eGFR nor mean follow-up eGFR were associated with sex.

In multiple regression, urine NGAL and serum albumin were identified as the independent predictors of eGFR changes and eGFR values based on mean serum creatinine during the follow-up (Table 3). When uACR and NGAL to creatinine ratio were included in the models instead of uncorrected urine albumin and NGAL concentrations, very similar results were obtained (beta ± SE for NGAL to creatinine ratio: −0.24 ± 0.11; *p* = 0.025 for final to baseline eGFR ratio as the dependent variable; −0.30 ± 0.10; *p* = 0.004 for final—baseline eGFR; and −0.12 ± 0.04; *p* = 0.005 for eGFR based on mean creatinine during the follow-up as the dependent variable, respectively). This results were observed despite the significant correlations of urinary NGAL concentration and NGAL/creatinine ratio with urine albumin and uACR.

**Figure 2:**
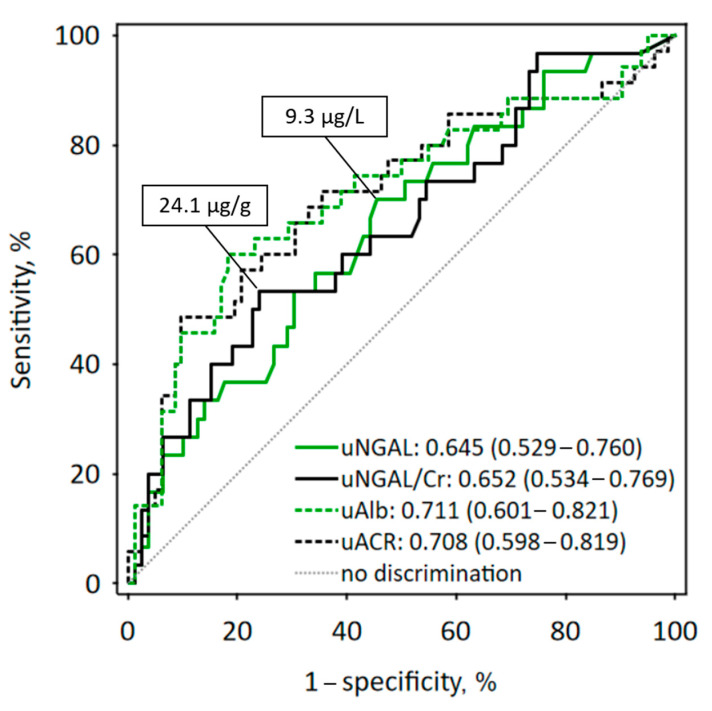
Receiver operating characteristic (ROC) curves for baseline urine NGAL concentration (uNGAL; solid green line) and urine NGAL/creatinine ratio (uNGAL/Cr; solid black line) in the prediction of ≥10% decrease of eGFR during the one-year follow-up. The cut-off values are reported on the graph. For comparison, ROC curves for urine albumin concentration (uAlb) and urine albumin/creatinine ratio are presented using dashed lines. The values of area under the ROC curves (AUC) are shown with 95% confidence intervals in brackets.

ROC curve analysis (Figure 2) showed a weak diagnostic accuracy of baseline urinary NGAL concentration and NGAL/creatinine ratio in the prediction of ≥10% decrease in eGFR values during the follow-up. However, the area under the ROC curve (AUC) values for urine NGAL concentration and NGAL/creatinine ratio were not significantly different from the AUC values obtained for urine albumin concentration and uACR (*p* = 0.7 in all comparisons). The cut-off values selected using maximum Youden index were 9.3 µg/L for urine NGAL concentration (associated with diagnostic sensitivity of 70% and specificity of 54%) and 24.1 µg/g for NGAL/creatinine (sensitivity 53% and specificity 76%), respectively.

We further analyzed the association between the baseline urinary NGAL and the changes in eGFR observed during the study in the subgroups of patients based on the time from transplantation: the correlations were weaker in the patients who were recruited between the end of the first and sixth year post-transplant as compared to those who had longer post-transplant history (Table 4). Moreover, in patients recruited more than six years post-transplantation, we observed significant correlation between urinary NGAL/creatinine (log-transformed) and eGFR at baseline (R = −0.28; *p* = 0.035).

### 3.5. Associations between Urinary NGAL and Persistent or Increasing Proteinuria

We analyzed the laboratory records of studied patients with respect to urine albumin or protein concentrations measured during the one-year follow-up. During the observation, proteinuria persisted or increased in 6 (15%) of 39 patients with A2 albuminuria and 15 (71%) of 21 patients with A3 albuminuria recorded at baseline. In the remaining patients, urine protein concentrations decreased during the observation. The patients with baseline uACR <30 mg/g did not develop increased proteinuria during the study. Among patients with persistent or increasing proteinuria, there were 6 (29%) recipients of second renal transplant, a significantly higher percentage as compared to the rest of the studied group (6 of 88; 7%; *p* = 0.004). Eleven (52%) of the 21 patients were diagnosed with glomerular disease versus 28 (32%) in the rest of the group (*p* = 0.08). Age, time from transplantation, number of donor/recipient HLA mismatches, pretransplant PRA, the use of induction therapy, or transplant ischemia times did not differ between the groups. Persistent proteinuria was associated with higher decline in eGFR during the observation (median final/baseline eGFR 85% versus 101%; *p* < 0.001). The 21 patients with persistent or increased proteinuria were characterized by significantly higher baseline urine NGAL concentrations: median (Q1; Q3) 23.0 (9.25; 45.2) versus 8.75 (3.95; 19.7) µg/L (*p* = 0.002) and NGAL/creatinine ratios: 35.1 (14.7; 84.9) versus 13.1 (5.91; 23.4) µg/g (*p* = 0.005).

### 3.6. Associations between Urinary NGAL and MMP 9-NGAL Complex and the Incidence of Bacterial Urinary Tract Infections during the Follow-Up Period

During the follow-up, bacterial urinary tract infections were diagnosed in 20 patients: four (14%) in the group that experienced ≥10% decrease in eGFR and 16 (20%) in the group with final eGFR >90% of initial value. The incidence of urinary tract infections did not differ significantly between the groups (*p* = 0.4). Also, eGFR values based on the mean serum creatinine during the follow-up did not differ between patients who developed urinary tract infections during the follow-up and those who did not (median 51 and 47 mL/min/1.73 m^2^, respectively; *p* = 0.9).

**Figure 3:**
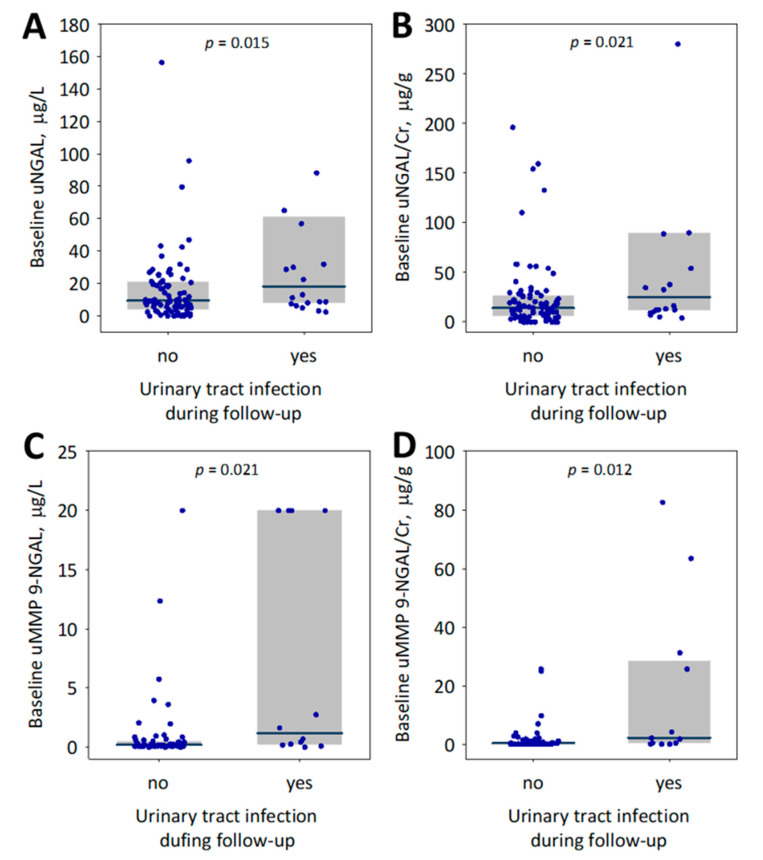
Baseline urine NGAL concentration (uNGAL; (**A**)), urine NGAL to creatinine ratio (uNGAL/Cr; (**B**)), urine MMP 9-NGAL complex concentration (uMMP 9-NGAL; (**C**)) and urine MMP 9-NGAL complex to creatinine ratio (uMMP 9-NGAL/Cr; (**D**)) among studied kidney transplant recipients who did or did not experience bacterial urinary tract infections during the follow-up period. Data are shown as median (central line), interquartile range (box) and raw data (points); *p*-values are given for the differences between groups. To increase readability of the graphs, we omitted the highest results of urine NGAL (528; 1275; 1401 µg/L; panel (**A**)) and NGAL/creatinine (1251; 1656; 2183 µg/g; panel (**B**)) obtained in three patients who experienced urinary tract infection during follow-up.

Baseline urine NGAL concentration, NGAL/creatinine ratio, MMP 9-NGAL complex and the complex to creatinine ratio were all significantly associated with bacterial urinary tract infections during the follow-up (Figure 3). The urinary tract infections were more common in women than in men (32% versus 9%, respectively; *p* = 0.002) while they were not associated with diabetes (*p* = 0.6) or immunosuppressive treatment modality (*p* > 0.4). In logistic regression analysis, log-transformed urinary NGAL concentration (odds ratio per unit change 3.46; 95% confidence interval 1.29–9.21; *p* = 0.012) or log-transformed NGAL/creatinine ratio (odds ratio per unit change 3.10; 95% confidence interval 1.20–7.97; *p* = 0.018) predicted urinary tract infections during the follow-up independently of sex. On the contrary, urinary concentration of MMP 9-NGAL complex was significantly associated with sex (median 0.162 in men and 0.552 in women; *p* < 0.001) and did not prove sex-independent predictor of urinary tract infections. Urinary albumin and uACR were not associated with urinary tract infections during the follow-up (*p* = 0.3 for both comparisons).

## 4. Discussion

In our sample of kidney transplant recipients recruited at least one year after the transplantation, without acute conditions diagnosed before and on enrollment, baseline urinary NGAL measured with the automated laboratory method significantly predicted the changes in eGFR values observed during the following year independently of other predictors, most importantly the baseline eGFR and albuminuria. Although the diagnostic accuracy of urine NGAL in the prediction of eGFR decline was low (the AUC of 0.65 in ROC curve analysis), it was statistically significant and did not differ significantly from the diagnostic accuracy of urine albumin. Higher baseline NGAL was also associated with persistent or increasing proteinuria during one-year observation of the studied patients. Moreover, higher baseline urinary NGAL and MMP 9-NGAL complex were observed in patients who developed urinary tract infections during the follow-up.

Although we recruited “clinically stable” patients, i.e., without any acute conditions (including infections and kidney injury) before and on enrollment, we observed significant changes in eGFR values during the follow-up: the final eGFR values were in the range of 40–170% of the initial values or −30 to +25 mL/min/1.73 m^2^. Serum creatinine concentrations (and thus GFR estimate) is dependent of non-renal factors, e.g., water balance, nutritional status, physical exercise, other metabolic factors, or diet [34]. Together with the laboratory imprecision, these factors lead to the variability in serum creatinine and eGFR that must be considered when monitoring the patients with chronic kidney disease. As stated in KDIGO guidelines for the care of kidney transplant recipients [1], because of within-subject variation of serum creatinine, a 25–50% increase of creatinine concentrations over baseline is predictive of the subsequent graft failure and should be considered an indication for graft biopsy (after excluding acute causes such as dehydration or blocked urine output). Considering the well-known inverse hyperbolic association between serum creatinine and GFR, the 50% increase in creatinine may be translated into roughly 25% decrease in GFR [35]. On the other hand, a decrease in GFR of 5 mL/min/1.73 m^2^ per year is considered a rapid progression of chronic kidney disease [33]. We believe that a 10% decrease in eGFR over a year should be clinically interpreted as a warning sign, despite the variability observed in clinical practice, and the causes of such a decrease should be sought.

According to the recent studies, chronic graft dysfunction is mainly caused by chronic antibody-mediated rejection, interstitial fibrosis, and tubular atrophy (IF/TA) induced by various chronic inflammatory processes, and BK polyoma virus (BKV) nephropathy. In protocol biopsies done in 1000 transplant recipients at 3, 12, 24, 48, and 60 months post-transplant, the signs of chronic rejection were detected in 17% of patients, and IF/TA in 30% [3]. The long-term function of kidney transplant and the risk of graft loss may be predicted based on serum creatinine and proteinuria at one year following kidney transplantation [36]. Moreover, anemia and high systolic blood pressure have been associated with higher risk of graft loss [36]. While overt proteinuria (detectable by urine dipstick) occurring in a kidney transplant recipient is a serious adverse predictor of graft survival, we need a non-invasive biomarker (or markers) that would allow detecting the injury at an earlier stage, before it leads to significant decrease in GFR or overt proteinuria.

Most studies evaluating urinary NGAL in kidney transplant recipients used NGAL concentrations measured early (hours till days) after transplantation procedure. Urinary NGAL measured between 6 and 48 h from the surgery predicted delayed graft function in most studies (although the diagnostic accuracy of serum/plasma NGAL in this context has been better in some studies) [26,37,38,39,40,41]. Consistently, urinary NGAL measured early post-transplant was shown to predict graft function at one year [39,41]. Recently, Maier et al. [42] assessed serum and urine NGAL in 170 kidney transplant recipients during the first week following transplantation and showed the best diagnostic utility of day 2 measurements for the delayed graft function. Both serum and urine NGAL concentrations on day 2 after transplantation predicted delayed graft function independently of serum creatinine and urine output; however, NGAL as an independent variable did not reach statistical significance in the models predicting graft loss or recipient’s death at 2 or 5 years post-transplant [42].

We were able to identify only several reports where NGAL has been measured in kidney transplant recipients after the peri-transplantation period. Ramirez-Sandoval et al. [27] measured several tubular biomarkers including urinary NGAL in kidney transplant recipients with acute kidney injury: the median time from transplantation was 3.5 years. The urinary concentrations of NGAL were significantly higher in kidney transplant recipients with AKI as compared to kidney transplant controls with normal eGFR and normal transplant histology. Moreover, urine NGAL/creatinine ratios were higher in patients with immunological rejection as compared to those with prerenal or other causes of acute kidney injury [27]. In a prospective observation of kidney transplant recipients admitted with AKI that occurred after median period of three years from the transplantation, high urinary NGAL (>210 ng/mL) at admission significantly predicted graft loss during subsequent year, independently of baseline eGFR, serum creatinine on admission and time from transplantation [29]. We cannot directly compare our results to that of Ramirez-Sandoval [27,29], as our group did not include patients with clinically detectable acute kidney injury on enrollment. However, their results indicate that urinary NGAL measurements performed years after transplantation may be diagnostically and prognostically useful. In another study, Kaufeld et al. [28] measured urinary NGAL concentration six weeks, three months and six months after kidney transplantation and compared the concentrations between patients with or without acute tubular injury as verified by protocol transplant biopsies done in the same time points. At six weeks, but not at six months, urinary NGAL was higher in patients with tubular injury as compared to those without injury; moreover, women and patients with urinary tract infections on the day of sample collection had higher NGAL concentrations [28]. Kaufeld et al. [28] used enzyme-linked immunosorbent assay for NGAL determination, and, although the total number of patients in the analysis was 140, the measurements in individual time points were available in smaller subgroups (44 patients with tubular injury and 23 patients without tubular injury at six weeks post-transplant; 55 and 16 at six months, respectively). The study design of Kaufeld et al. [28] differed from ours in several aspects (much longer times from transplantation in our patients, automated method of NGAL determination in our study, and no protocol biopsies in our center), thus, the results cannot be directly compared. Most importantly, we were not able to verify the association between morphological changes in kidney and NGAL excretion. However, the association of NGAL with urinary tract infections was shown in both studies. In 2015, Cassidy et al. [30] measured NGAL as one of several urine biomarkers in 34 patients recruited more than one year after kidney transplantation and analyzed the measured concentrations in the context of histologically proven chronic fibrosis and a similar number of kidney transplant recipients with normal renal function. They observed significantly higher urinary NGAL concentrations in patients with chronic allograft nephropathy [30]. Our results remain in line with the findings of Cassidy et al. [30], although the methods of NGAL determination differed (Cassidy et al. used Western-blot and ELISA). Lacquaniti et al. [31] recruited 84 renal transplant recipients one year or more after renal transplantation. They observed higher baseline serum and urine NGAL concentrations (measured by ELISA) in the patients in whom serum creatinine concentrations doubled or who progressed to end-stage kidney failure during five years’ observation [31]. Urine NGAL above 71.8 µg/L was predictive of transplant function worsening during the observation (AUC of 0.889) [31]. This cut-off value is higher as compared to our results; however, the “worsening of kidney function” was more severe in the study of Lacquaniti et al. [31]; still, our results are in line with the findings. Finally, Schaub et al. [43] measured urinary concentrations of several renal tubular markers (retinol-binding protein, α1-microglobulin and NGAL) in kidney transplant recipients with median time from transplantation >90 days, who underwent either protocol or indication graft biopsy and were divided into four groups based on the biopsy findings regarding tubular pathology. Patients with clinical tubulitis and other tubular pathology had higher concentrations of measured proteins including NGAL. Moreover, subclinical tubulitis was associated with non-significantly (*p* = 0.06) higher NGAL as compared to transplant recipients with normal histology [43]. The results of Schaub et al. [43] show that urinary NGAL measured late after the transplantation (median time from transplantation was 158 days in the group with clinical tubulitis and 1163 days in patients with other tubular pathology) is indicative of tubular pathology in kidney transplant recipients.

In non-transplanted chronic kidney disease patients, higher urinary NGAL was associated with a chronic decline in kidney function [21,44]. Moreover, in renal transplant recipients, the presence of tubular proteinuria with increase in urinary concentrations of retinol-binding protein or α1-microglobulin (i.e., low molecular weight proteins that are normally reabsorbed in proximal tubule) after the first year post kidney transplantation has been associated with decline in transplant function during long-term follow-up [45,46], which is in line with our results.

While the NGAL monomer has been associated with tubular injury, and neutrophils seem the main source of NGAL dimer in urine [13,16,47], the complex of MMP 9 (gelatinase B) with NGAL has not been extensively studied in the context of kidney diseases. The 135 kDa complex of MMP 9 (gelatinase B) with NGAL has been originally described and purified from neutrophils isolated from blood and activated with phorbol myristate acetate. Later, the „high molecular weight metalloproteinase”, present in urine of patients with cancer, has been characterized as a 125 kDa complex of MMP 9 and NGAL [48]. The MMP 9-NGAL complex has been found in urine of breast cancer patients [49]. According to Yan et al. [48], the complex may be formed in urine from NGAL and MMP 9 that are separately filtered or secreted into urine. Alternatively, the neutrophils present in urine may be a source of the MMP 9-NGAL complex. In our sample of kidney transplant recipients, the latter is consistent with higher concentrations of the MMP 9-NGAL complex among patients who subsequently developed bacterial urinary tract infections as well as in women, more prone to urinary tract infections. Previously, the MMP 9-NGAL complex has been detected in dogs with pyuria and azotemia [50]. Of note, NGAL has been shown to prevent MMP 9 from proteolysis and a complex of MMP 9 with NGAL found in human urine has been shown to exert the gelatinase enzymatic activity [48,49]. This may have pathophysiological implications in kidney disease, as matrix metalloproteinases play a role in IF/TA affecting the transplanted kidney [51,52]. We measured MMP 9-NGAL complex in a subgroup of 77 patients, of whom only 19 had decreasing eGFR, and it is possible that our study was underpowered to find a weak association of the complex urinary concentrations with transplant function.

In our study, in addition to urinary NGAL, serum albumin and uric acid were also associated with the changes in eGFR and mean eGFR during the follow-up. Serum albumin is an indicator and predictor of poor nutritional status in kidney disease, moreover, it is a known negative acute phase protein [53,54]. The association of low serum albumin concentrations with decreasing eGFR and lower mean eGFR in our patients may reflect subclinical inflammatory states or worse nutritional status of the patients with subsequently decreasing kidney function. Of note, NGAL has been associated with low-grade inflammation in kidney disease [55,56]. Serum uric acid has been previously shown to predict adverse outcomes in kidney transplant patients [57].

Our study has several limitations: first, although we included more than 100 patients, the decrease in kidney function was detected in below one third of them, resulting in limited number of such patients. Furthermore, the data on donor/recipient HLA mismatches and PRA were available in a subgroup of patients, and we were not able to report the data on donor-specific antibodies, as these started to be measured in Poland only recently. The causes of decline in transplant function were not verified in histopathological examination because the protocol biopsies are not practiced in our center, and because there were patients who did not agree to indication biopsy. A longer follow-up time would allow studying the association of baseline NGAL with subsequent graft loss, a more robust end-point as compared to the one chosen in our study. In our group only two patients started dialysis treatment due to the end-stage graft failure. However, it was our aim to seek for the predictors of less severe decline in transplant function.

The strength of our study is that we measured urinary NGAL with a robust automated method that may be used in routine clinical practice (and indeed it is available in many centers). Although several studies show the diagnostic utility of urine NGAL for the prediction of adverse outcomes in kidney transplant recipients, the lack of standardization between the assays hinders the use of NGAL in clinical practice. We believe that the automated methods should be used in the studies if we want to translate the studies’ results into practice.

In summary, our study indicates that the urinary NGAL and NGAL to creatinine ratio predicts changes in kidney graft function over subsequent year in kidney transplant recipients with long-term functioning graft. The association between urinary NGAL concentration and the follow-up graft function is moderate but independent of baseline eGFR and albuminuria. To which extent this prognostic utility of NGAL may be associated with the prediction of urinary tract infections, remains to be elucidated. Based on our observation, we may hypothesize that a high urinary NGAL measured as an additional marker in a clinically stable kidney transplant recipient should be interpreted as a warning sign, leading to detailed evaluation of a patient in search of either transient or chronic causes of graft dysfunction, or urinary tract infection. Low urinary NGAL is associated with a low risk of kidney graft dysfunction over a subsequent year. However, this clinical hypothesis must be verified in larger studies, at best involving the detailed characterization of graft state with the use of protocol biopsies.

## Figures and Tables

**Table 1:** Baseline clinical characteristics of patients according to eGFR changes during follow-up.

Characteristic	eGFR at the End of Follow-Up		*p*
	≤90% of Initial Value (*n* = 30)	>90% of Initial Value (*n* = 79)	
Mean age ± SD, years	54 ± 14	54 ± 13	0.8
Male sex, *n* (%)	18 (60)	48 (73)	0.9
Median time from transplantation (Q1; Q3), years	8 (5; 14)	7 (3; 13)	0.5
Primary cause of kidney disease			
glomerular diseases, *n* (%)	12 (40)	27 (34)	0.9
tubulointerstitial diseases, *n* (%)	4 (13)	13 (16)	
vascular diseases, *n* (%)	1 (3)	5 (6)	
cystic/congenital diseases, *n* (%)	3 (10)	12 (15)	
unknown, *n* (%)	10 (33)	22 (28)	
First transplant, *n* (%)	25 (83)	72 (91)	0.2
Second transplant, *n* (%)	5 (17)	7 (9)	
Deceased donor, *n* (%)	30 (100)	78 (99)	1.0
Median number of HLA mismatches (Q1; Q3) *	3 (2; 4)	3 (3; 4)	0.8
Median peak pretransplant PRA (Q1; Q3), % *	10 (0; 30)	0 (0; 3)	0.045
Median last pretransplant PRA (Q1; Q3), % *	0 (0; 10)	0 (0; 0)	0.1
Induction therapy, *n* (%)	3 (10)	11 (19)	0.9
no data, *n* (%)	13 (43)	21 (27)	
Median cold ischemia time (Q1; Q3), min **	1320 (1170; 1566)	1100 (840; 1470)	0.06
Median warm ischemia time (Q1; Q3), min **	35 (29; 50)	32 (27; 40)	0.3
Delayed graft function, *n* (%)	11 (37)	21 (27)	0.5
no data, *n* (%)	10 (33)	19 (24)	
Immunosuppression			
glucocorticoids, *n* (%)	30 (100)	76 (96)	0.6
MMF or MPA, *n* (%)	28 (93)	74 (94)	0.9
cyclosporine, *n* (%)	7 (23)	22 (28)	0.6
tacrolimus, *n* (%)	21 (70)	53 (67)	0.8
mTOR inhibitor, *n* (%)	2 (7)	5 (7)	0.9
Diabetes, *n* (%)	8 (27)	13 (16)	0.2
Hypoglycemic agents			
oral, *n* (%)	4 (13)	9 (11)	0.8
insulin, *n* (%)	4 (13)	5 (6)	0.2
Use of RAA blockers, *n* (%)	11 (37)	35 (44)	0.5
Median daily diuresis (Q1; Q3), mL	2500 (2000; 3000)	2500 (2000; 3000)	0.4
Mean BMI ± SD, kg/m^2^	25 ± 5	27 ± 5	0.2
Mean systolic pressure ± SD, mmHg	138 ± 15	133 ± 13	0.1
Mean diastolic pressure ± SD, mmHg	84 ± 11	83 ± 10	0.9

* Available in 46 patients (14 with decreasing eGFR and 32 with non-decreasing eGFR) who underwent transplantation procedure in University Hospital, Kraków, Poland; ** Available in 80 patients (19 with decreasing eGFR and 61 with non-decreasing eGFR). BMI, body mass index; eGFR, estimated glomerular filtration rate; MMF, mycophenolate mofetil; MPA, mycophenolic acid; mTOR, mammalian target of rapamycin; PRA, panel reactive antibodies; Q1, first quartile; Q3, third quartile; RAA, renin-angiotensin-aldosterone system; SD, standard deviation.

**Table 2:** Baseline results of laboratory tests according to eGFR changes during follow-up. Data are shown as mean ± SD or median (Q1; Q3).

Laboratory Test	eGFR at the End of Follow-Up		*p*
	≤90% of Initial Value (*n* = 30)	>90% of Initial Value (*n* = 79)	
Urine albumin, mg/L	165 (20; 731)	21 (8; 67)	0.001
uACR, mg/g	194 (28; 1066)	26 (11; 159)	0.001
Serum creatinine, µmol/L	142 (111; 205)	127 (97; 168)	0.09
eGFR (CKD-EPI_Cr_), mL/min/1.73m^2^	38 (31; 54)	49 (38; 70)	0.038
Urine NGAL, µg/L	17.8 (8.0; 30.0)	8.9 (4.0; 21.6)	0.020
Urine NGAL/creatinine, µg/g	25.2 (9.8; 58.5)	13.2 (5.6; 22.7)	0.015
Urine MMP 9-NGAL complex, µg/L *	0.255 (0.141; 0.584)	0.216 (0.13; 0.681)	0.8
Urine MMP 9-NGAL/creatinine, µg/g *	0.35 (0.21; 1.71)	0.39 (0.23; 1.29)	0.9
Hemoglobin, g/dl	12.4 (11.1; 13.4)	13.2 (12.6; 14.5)	0.005
White blood count, ×10^3^/µL	7.50 (5.80; 8.38)	7.07 (5.97; 8.51)	0.8
Platelet count, ×10^3^/µL	196 ± 53	211 ± 56	0.2
Triglycerides, mmol/L	1.64 (1.28; 2.12)	1.58 (1.21; 2.12)	0.9
Total cholesterol, mmol/L	5.17 (4.20; 5.66)	4.91 (4.21; 5.73)	0.7
Uric acid, mmol/L	378 (342; 431)	379 (334; 431)	0.9
C-reactive protein, mg/L	1.84 (1.00; 4.44)	1.41 (1.00; 2.99)	0.5
Serum albumin, g/L	42 (40; 44)	44 (43; 46)	0.002
Glucose, mmol/L	5.55 (5.16; 6.06)	5.44 (5.07; 6.02)	0.7

* Available in 77 patients (19 with decreasing eGFR and 55 with non-decreasing eGFR). eGFR, estimated glomerular filtration rate; CKD-EPI_Cr_ [33], Chronic Kidney Disease—Epidemiology Collaboration equation based on serum creatinine; NGAL, neutrophil gelatinase-associated lipocalin; MMP 9, matrix metalloproteinase 9; uACR, urinary albumin to creatinine ratio.

**Table 3:** Simple correlations and multiple models showing the associations between studied variables and the changes in eGFR values over the follow-up period (defined either as the follow-up to baseline eGFR ratio or as the difference between follow-up and baseline eGFR) and mean eGFR during the follow-up. The multiple models were adjusted for time from kidney transplantation.

Independent Variable	Dependent Variable											
	Final/Baseline eGFR				Final—Baseline eGFR				eGFR Based on Mean Creatinine during Follow-Up			
	Simple Correlation		Multiple Regression		Simple Correlation		Multiple Regression		Simple Correlation		Multiple Regression	
	R	*p*	beta ± SE	*p*	R	*p*	beta ± SE	*p*	R	*p*	beta ± SE	*p*
Baseline eGFR	−0.09	0.3	−0.04 ± 0.11	0.7	−0.22	0.020	−0.13 ± 0.11	0.2	0.92	<0.001	0.98 ± 0.04	<0.001
log (uAlb)	−0.26	0.006	−0.08 ± 0.12	0.5	−0.16	0.08	−0.01 ± 0.11	0.9	−0.36	<0.001	0.04 ± 0.04	0.4
log (uACR)	−0.24	0.012	not included		−0.14	0.1	not included		−0.36	<0.001	not included	
log (uNGAL)	−0.31	0.001	−0.22 ± 0.10	0.039	−0.32	0.001	−0.28 ± 0.10	0.006	−0.19	0.045	−0.11 ± 0.04	0.008
log (uNGAL/Cr)	−0.31	0.002	not included		−0.29	0.003	not included		−0.22	0.022	not included	
log (uric acid)	0.21	0.028	0.15 ± 0.10	0.2	0.19	0.051	0.08 ± 0.10	0.4	−0.33	0.001	0.10 ± 0.04	0.014
Serum albumin	0.34	<0.001	0.24 ± 0.10	0.018	0.36	<0.001	0.29 ± 0.10	0.004	0.06	0.6	0.11 ± 0.04	0.007

eGFR, estimated glomerular filtration rate; uAlb, urinary albumin; uACR, urine albumin to creatinine ratio; uNGAL, urine neutrophil gelatinase-associated lipocalin; uNGAL/Cr, urine NGAL to creatinine ratio; SE, standard error.

**Table 4:** Correlations between the baseline urine NGAL concentrations (uNGAL) and NGAL to creatinine ratios (uNGAL/Cr) and the changes in eGFR observed during the study as well as the eGFR based on mean serum creatinine during the follow-up.

Variable	Time from Transplantation at the Start of the Study			
	1–6 Years (*n* = 49)		7–22 Years (*n* = 60)	
	uNGAL	uNGAL/Cr	uNGAL	uNGAL/Cr
Final/baseline eGFR	R = −0.28; *p* = 0.06	R = −0.27; *p* = 0.07	R = −0.34; *p* = 0.008	R = −0.36; *p* = 0.007
Final–baseline eGFR	R = −0.34; *p* = 0.023	R = −0.29; *p* = 0.05	R = −0.31; *p* = 0.020	R = −0.31; *p* = 0.019
eGFR based on mean creatinine during follow-up	R = −0.08; *p* = 0.6	R = −0.06; *p* = 0.7	R = −0.29; *p* = 0.029	R = −0.35; *p* = 0.007

## Data Availability

The data are available from the corresponding author upon reasonable request.
